# Supplementary material for: Laminin α4 Expression in Human Adipose Tissue Depots and Its Association with Obesity and Obesity Related Traits
Source: Biomedicines. 2023 Oct 17;11(10):2806. doi: 10.3390/biomedicines11102806 (PMC10604865; doi:10.3390/biomedicines11102806)
Supplement: Supplementary file 1 [file biomedicines-11-02806-s001.zip › Suppl_Figures_Captions.pdf]

Supplementary Material

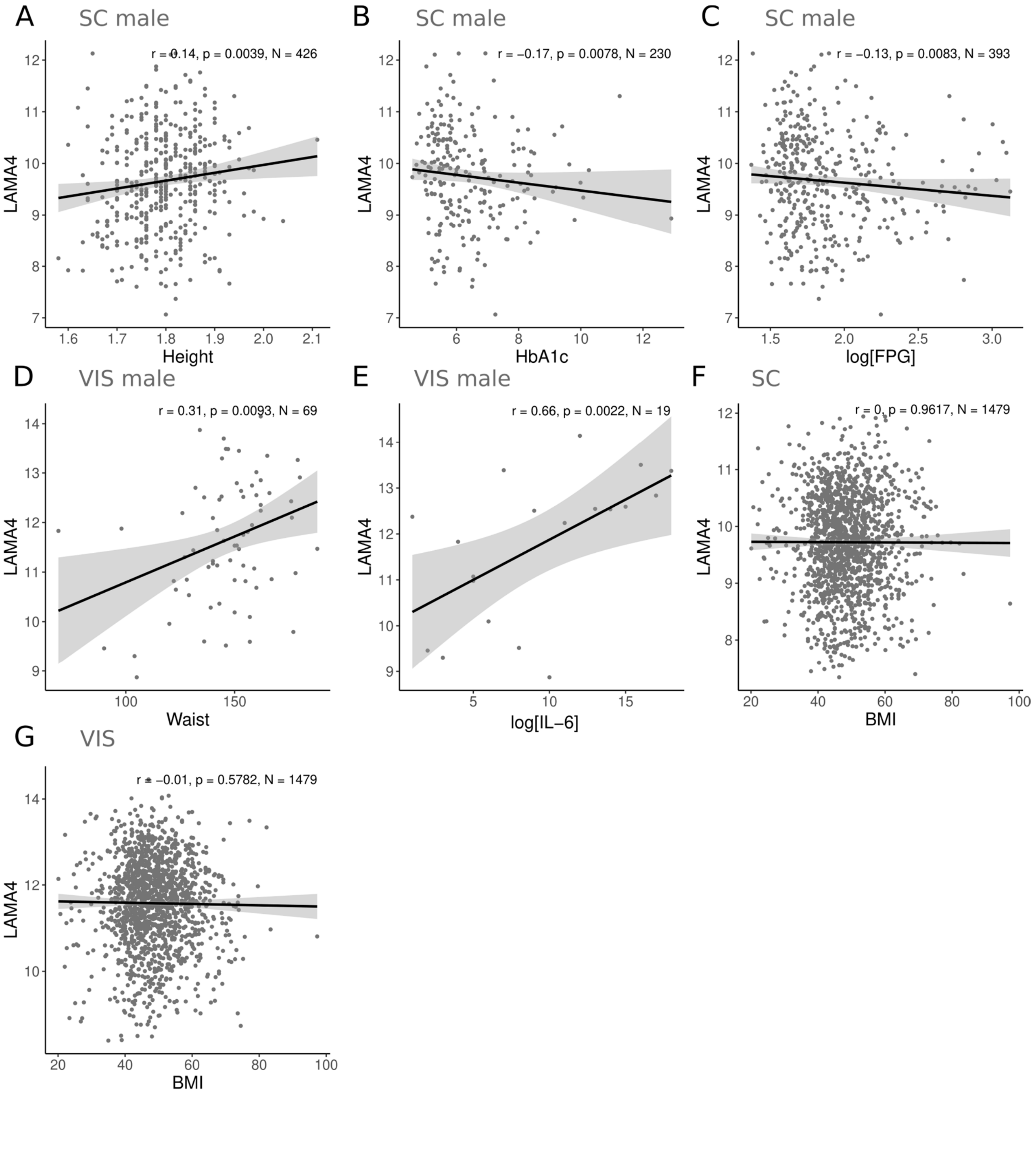

Supplementary Figure S1 Correlation of *LAMA4* expression with clinical parameters within the cross-sectional cohort.

In male SC AT, *LAMA4* correlates with **A)** height (ρ = 0.14, *P* = 0.004, N = 426), **B)** HbA_{1C} (ρ = -0.17, *P* = 0.008, N = 230) and **C)** FPG (ρ = -0.13, *P* = 0.008, N = 393). In male VIS AT, *LAMA4* positively correlates with **D)** waist (ρ = 0.31, *P* = 0.0093417, N = 69) and **E)** IL-6 (ρ = 0.66, *P* = 0.002, N = 19). Associations of *LAMA4* with BMI could not be found in both **F)** SC AT (ρ = -0.001, *P* = 0.96, N = 1,479) and **G)** VIS AT (ρ = -0.01, *P* = 0.57, N = 1,479). P-values are not adjusted for multiple testing. AT: adipose tissue; BMI: body mass index; FPG: fasting plasma glucose; HbA_{1C}: hemoglobin A_{1c}; IL-6: interleukin 6; SC: subcutaneous; VIS: visceral

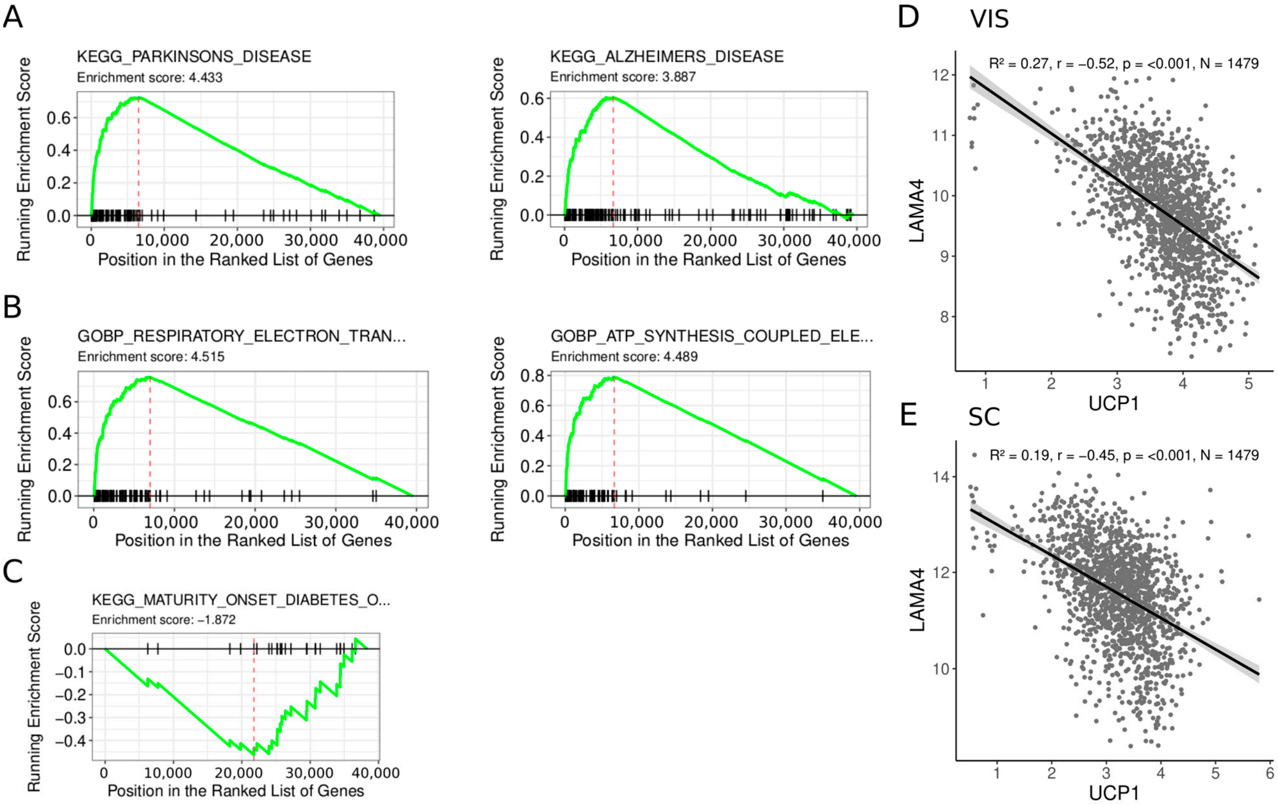

Supplementary Figure S2 Gene set enrichment analysis and correlation of *LAMA4* within the cross-sectional cohort. Gene set enrichment analysis (GSEA) of co-expressed genes with *LAMA4* expression in VIS AT shows enrichment for pathways related to **A)** neurodegenerative diseases (using the Kyoto Encyclopedia of Genes and Genomes (KEGG) and **B)** metabolism and energy expenditure (using the Gene Ontology database for biological processes). **C)** Maturity onset diabetes KEGG pathway is significantly underrepresented in SC AT, analyzed with GSEA. Correlation analysis reveals associations between *LAMA4* and *UCP1* expression in **D)** VIS AT and **E)** SC AT, analyzed with Spearman's correlation coefficient. GOBP: gene ontology biological process; KEGG: Kyoto Encyclopedia of Genes and Genomes; UCP1: uncoupling protein-1

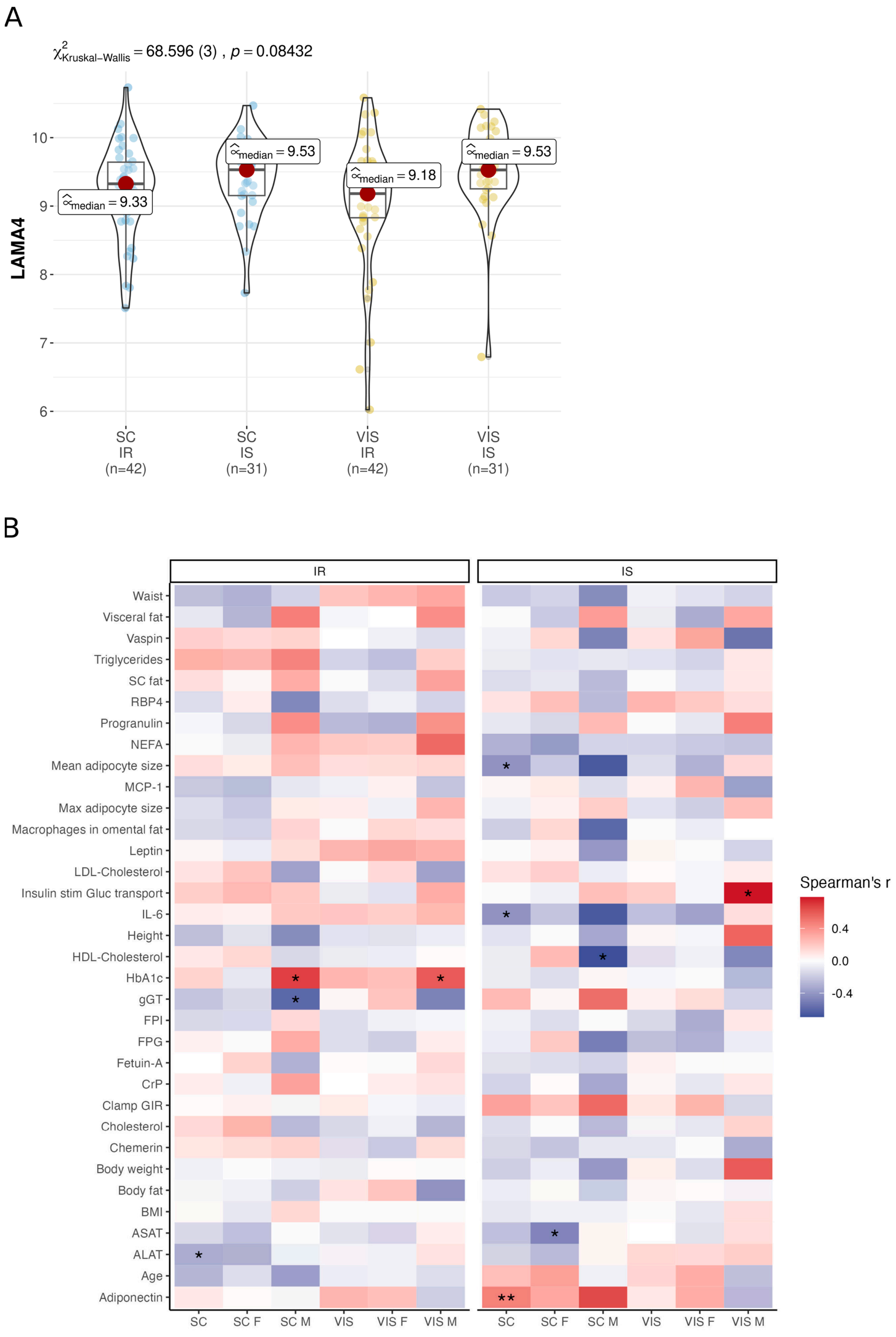

A
$\chi^2_{\text{Kruskal-Wallis}} = 68.596\ (3)\ ,\ p = 0.08432$
LAMA4
10
9
8
7
6
$\widehat{\mu}_{\text{median}} = 9.33$
$\widehat{\mu}_{\text{median}} = 9.53$
$\widehat{\mu}_{\text{median}} = 9.18$
$\widehat{\mu}_{\text{median}} = 9.53$
SC IR (n=42)
SC IS (n=31)
VIS IR (n=42)
VIS IS (n=31)
B
IR
IS
Waist
Visceral fat
Vaspin
Triglycerides
SC fat
RBP4
Progranulin
NEFA
Mean adipocyte size
MCP-1
Max adipocyte size
Macrophages in omental fat
Leptin
LDL-Cholesterol
Insulin stim Gluc transport
IL-6
Height
HDL-Cholesterol
HbA1c
gGT
FPI
FPG
Fetuin-A
CrP
Clamp GIR
Cholesterol
Chemerin
Body weight
Body fat
BMI
ASAT
ALAT
Age
Adiponectin
SC
SC F
SC M
VIS
VIS F
VIS M
Spearman's r
0.4
0.0
-0.4

Supplementary Figure S3: Analysis of *LAMA4* gene expression and correlation within the metabolic healthy and unhealthy obesity cohort. A) Comparison of *LAMA4* expression between insulin resistant (IR) and insulin sensitive (IS) subjects in SAT and VAT, analyzed with Kruskal-Wallis one-way ANOVA and Dunn's test for pairwise comparisons, corrected for multiple comparisons using the Hommel method. **B)** Correlation between *LAMA4* gene expression and body composition/metabolic parameters in SC and VIS AT, analyzed with Spearman's correlation coefficient. ALAT: Alanine transaminase; ASAT: Aspartate transaminase; BMI: body mass index; GIR: glucose infusion rates; CrP: C-reactive protein; F: female; FPG: fasting plasma glucose; FPI: fasting plasma insulin; gGT: Gamma-glutamyltransferase; HOMA-IR: Homeostatic Model Assessment for Insulin Resistance; IR: insulin resistant; IS: insulin sensitive; MCP-1: Monocyte chemoattractant protein-1; M: male; NEFA: nonesterified fatty acids; RBP4: Retinol binding protein 4; SC: subcutaneous; WHR: Waist hip ratio; VIS: visceral

Supplemental Table S1: Correlation analysis of *LAMA4* gene expression within the cross-sectional cohort. This table shows the spearman coefficients of *LAMA4* expression correlations with clinical parameters within the cross-sectional cohort. Correlation analyses were performed in both SC and VIS AT for all individuals and divided by gender. P-values are reported before and after adjustment for multiple testing with the Hommel method. N refers to the number of individuals for whom clinical data was available. ALAT: Alanine transaminase; ASAT: Aspartate transaminase; BMI: body mass index; CrP: C-reactive protein; FPG: fasting plasma glucose; FPI: fasting plasma insulin; HbA_{1C}: hemoglobin A_{1c}; HDL: High Density Lipoprotein; HOMA-IR: Homeostatic Model Assessment for Insulin Resistance; IL-6: interleukin 6; LDL: Low Density Lipoprotein; NEFA: nonesterified fatty acids; SC: subcutaneous; WHR: Waist hip ratio; VIS: visceral

Supplementary Table S2: Gene ontology gene set enrichment analysis within the cross-sectional cohort. Gene set enrichment analysis results using the Gene Ontology database for biological processes show enriched sets of *LAMA* co-expressed genes in both SC and VIS AT within the cross-sectional cohort. Reported enrichment scores are raw values as well as normalized over gene set size. Significance is shown as p-values and adjusted p-values after correction for gene set size and multiple testing with FDR.

Supplementary Table S3: Kyoto Encyclopedia of Genes and Genomes gene set enrichment analysis within the cross-sectional cohort. Gene set enrichment analysis results using the Kyoto Encyclopedia of Genes and Genomes show enriched sets of *LAMA* co-expressed genes in both SC and VIS AT within the cross-sectional cohort. Reported enrichment scores are raw values as well as normalized over gene set size. Significance is shown as p-values and adjusted p-values after correction for gene set size and multiple testing with FDR.

Supplementary Table S4: Correlation analysis of *LAMA4* gene expression within the bariatric surgery cohort. This table shows the spearman coefficients of *LAMA4* expression correlations with clinical parameters within the bariatric surgery cohort. Correlation analyses were

performed in both SC and VIS AT at both pre- and post-surgery timepoints for all individuals and divided by gender. P-values are reported before and after adjustment for multiple testing with the Hommel method. N refers to the number of individuals for whom clinical data was available.

Supplementary Table S5: Correlation analysis of *LAMA4* gene expression within the cohort for distinguishing metabolically healthy versus unhealthy obesity. This table shows the spearman coefficients of *LAMA4* expression correlations with clinical parameters within the cohort for distinguishing metabolically healthy versus unhealthy obesity. Correlation analyses were performed in SC and VIS AT in both insulin sensitive and insulin resistant individuals and divided by gender. P-values are reported before and after adjustment for multiple testing with the Hommel method. N refers to the number of individuals for whom clinical data was available. GIR: glucose infusion rates; gGT: Gamma-glutamyltransferase; Insulin Resistance; IR: insulin resistant; IS: insulin sensitive; MCP-1: Monocyte chemoattractant protein-1; RBP4: Retinol binding protein 4
